# Health-related quality of life of the rural-China left-behind children or adolescents and influential factors: a cross-sectional study

**DOI:** 10.1186/s12955-015-0220-x

**Published:** 2015-02-27

**Authors:** Yun Huang, Xiao-Ni Zhong, Qing-Ying Li, Dan Xu, Xuan-Lin Zhang, Chao Feng, Guo-Xiu Yang, Yun-Yun Bo, Bing Deng

**Affiliations:** Department of Health Statistics, School of Public Health and Management, Chongqing Medical University, 1 Yixueyuan Road, Yuzhong District Chongqing, 400016 China

**Keywords:** Left-behind children, Left-behind adolescents, HRQoL, PedsQL, Rural area, China

## Abstract

**Background:**

Due to sustained export of labor service, the left-behind children/ adolescents in rural areas of China have become a group that can no longer be neglected. However, even up to this day, little is known about the health-related quality of life (HRQoL) of the left-behind children/adolescents, particularly in Midwest China. This study aims at investigating their living condition and analyzing the influential factors of their HRQoL.

**Methods:**

A cross-sectional study based on households was conducted and 1363 children or adolescents from rural areas of 6 provinces in China, among whom 608 were left-behind and 755 were non-left-behind, were enrolled in a multistage sampling. HRQoL was revealed using the Pediatric Quality of Life Inventory (PedsQL). Differences in scores were analyzed using rank sum tests, and multivariate analyses were conducted with multiple linear regression.

**Results:**

There was a total of 608 (44.61%) left-behind children or adolescents, and they scored significantly lower in terms of the HRQoL synthesis scores (F = 6.14, P < 0.05), Physical Functioning (H = 33.18, P < 0.05), Emotional Functioning (H = 24.99, P < 0.05) and Social Functioning (H = 12.24, P < 0.05), compared with the non-left-behind. Multiple linear regressions indicated that age and mother’s final academic qualification were in positive correlation with the HRQoL of the left-behind children, while mother’s longer migrant working time and less frequent visits, and being reared by uncle/aunt etc., were potential risk factors for the left-behind children.

**Conclusions:**

The HRQoL scores of left-behind children or adolescents were significantly lower than those of their counterparts both in the physical and the psychological domains. Influential factors should be considered when relevant policies are being made and intervening practices are being undertaken in the future, so as to improve the HRQoL of the left-behind children or adolescents.

**Electronic supplementary material:**

The online version of this article (doi:10.1186/s12955-015-0220-x) contains supplementary material, which is available to authorized users.

## Background

As economy recovers, the existence of left-behind children and adolescents in rural area has become a hot button in China. *Left-behind children* or *adolescents* refers to children who are under 18, and are left behind at their original residence for at least six months by one or both parents who migrate to other place to work [1]. Such parents, known as “migrant workers”, may live and work in the city for years without access to social services such as elementary education (for children), public medical security, and housing subsidy, and have to leave their children at their original residence [2]. Since the late 1970s, lots of rural migrants have been swarming into urban areas to seek employment opportunities, which has led to the sharp increase of the left-behind children or adolescents in Mainland China.

A national survey of the left-behind children in rural areas conducted by China Women’s Federation in 2013 revealed that near 61.03 million children or adolescents in Mainland China were left at home, accounting for approximately 21.88% of the children and adolescents in China, and 46.74% of the left-behind group had migrating parents. The national survey also found that the left-behind group showed an uneven distribution in China, mainly gathering in the mid-west provinces on the labor-service exporting side such as Sichuan Province, Henan Province, Hunan Province, and Anhui Province, with Sichuan and Hunan respectively occupying 11.34% and 7.13% of the total number. In rural areas of Chongqing Municipality, Sichuan Province, Hunan Province, Jiangxi Province, more than half of the children or adolescents were left-behinds [3].

Meanwhile, the physical and /or the psychological problems of this group have aroused extensive concerns. United Nations Children’s Fund (UNICEF) recommended that strengthened academic research be conducted to fully understand the effects of parents’ migration on their children or adolescents left behind [4]. The left-behind children, suffering from lower dietary diversity, higher prevalence of growth retardation, and higher incidence rate of injuries and anemia, were of an inferior health status [5]. Many researchers said that prolonged separation from father and/or mother might cause a substantial psychological burden on the left-behind children or adolescents, in other words, they were subject to high anxiety level [6],stress, fear [7] etc.. Although the Chinese government has promulgated laws and regulations to protect the right of this group, it remains difficult for the left-behind group to grow up in as healthy and all-sided ways as their counterparts.

More and more people have come to pay attention to this group, but researches into the left-behind children or adolescents have usually only focused on one aspect, either physical or psychological. Few studies have considered using a comprehensive indicator to evaluate the status of this group, and only one study conducted in Shandong province in East of China revealed that the left-behind children showed poorer Health-related quality of life (HRQoL) than their counterparts due to psycho-social dysfunction [8]. Health-related quality of life (HRQoL) is a good instrument in evaluating an individual’s physical and psychological status, which includes the status of physical, psychological, and social interactions of a person’s subjective assessments [9]. In 1997, WHO proposed the definition of HRQoL as “individual’s perception of their position in life in the context of the culture and value systems, in which they live and in relation to their goals, expectations, standards and concerns”. HRQoL has gradually become an important practical health outcome measurement in health services research and widely recognized as a useful indicator of current use to measure the level of health [10,11].

Moreover, no study appears to have investigated HRQoL of the left-behind children or adolescents in Midwest China, which leaves a tremendous gap in our understanding of both the physical and psychological status of this group. Not only China, but also many labor-exporting countries of East Asia such as Philippines, Indonesia, Thailand are confronted with this inevitable issue [12,13]. Near 2-3% children were left behind in Indonesia, and The Philippines, with a doubled ratio, has reached an amount of some two million. The left-behind children issue is a hot topic in the international context as well.

In this study, we first investigated the HRQoL of the left-behind children or adolescents and their counterparts— the ‘non-left-behind’— by a scale known as The Pediatric Quality of Life Inventory Measurement Models (PedsQL™), and then analyzed the influential factors of the stranded children’s/adolescents’ HRQoL. The purposes of this study are: (1) to assess whether the left-behind children and adolescents show lower HRQoL than the non-left-behind and the correlated factors of influence; (2) to provide the basis for developing more appropriate interventions and related public policies for the left-behind children and adolescents.

## Method

### Participants and settings

We investigated the left-behind children or adolescents between 2 and 18 in rural areas of six provinces (Chongqing Municipality, Sichuan Province, Jiangxi Province, Hunan Province, Inner Mongolia Autonomous Region and Xinjiang Uygur Autonomous Region) in Mainland China. The left-behind children/adolescents are individuals who were left behind at their original residences, while one or both parents migrate to other places to work and have not been living together with them for at least six months. Their counterparts are children/adolescents between 2 and 18 who live with their parents at their original residences. To make the counterparts comparable, all the non-left-behind were recommended by the left-behind children or adolescents of similar ages in the same village.

The formula for sample size calculation of the left-behind group is shown below:

- $$ N={\left[\frac{Z_{\upalpha /2}*\upsigma}{\delta}\right]}^2={\left[\frac{1.96*8.27}{1.00}\right]}^2=263 $$
- *Z*_*α*/2_ = Standard normal variate (at 5% type 1 error (P < 0.05) it is 1.96). P values are considered significant below 0.05, hence 1.96 is used in the formula.
- *σ* = Expected standard deviation for left-behind children or adolescents based on previously published studies [8], hence 8.27 is used in the formula.
- *δ* = Absolute error or precision taken by researcher based on previously published studies [8], hence 1.00 is used in the formula.

Our sample size for the left-behind children and adolescents was doubled to 526 because we were to use snowball sampling.

According to the regional differences and the proportion of the floating population in Midwest China, children aged from 2 to 18 were recruited by conducting a snowball sampling method in Chongqing Municipality, Sichuan Province, Jiangxi Province, Hunan Province, Inner Mongolia Autonomous Region and Xinjiang Uygur Autonomous Region, among which Jiangxi Province, Hunan Province, Chongqing Municipality, Sichuan Province had higher emigration rate in China, with Jiangxi Province and Hunan Province at the highest level accounting for 7.8‰ and 7.0‰respectively, while Inner Mongolia Autonomous Region and Xinjiang Uygur Autonomous Region were of middle-ranking emigration rate (2.0-3.5‰) but with many minority inhabitants [14]. The Women’s Federations of these six provinces recommended 2–4 villages with a large number of the left-behind children/adolescents from each province (actually, the latest data about detailed emigration rate of these six provinces are unavailable), and finally 14 villages were selected. The research has been approved by the Ethics Committee of Chongqing Medical University (Chongqing, China) [Additional file 1]. All participants have provided written informed consents [Additional file 2].

### Measurement

The questionnaires, which consist of general social demographic characteristics and the Pediatric Quality of Life Inventory TM 4.0 (PedsQL) were revised and verified carefully by relevant experts. The general social demographic characteristics such as gender, age, caregiver, and educational level of the parents and caregiver(s) as well as the parents’ visit interval etc., have been included in the survey.

The Pediatric Quality of Life Inventory (PedsQL), which was invented by Professor James W. Varni in Children’s Hospital and Health Center, San Diego, California, is one of the most promising HRQoL measurements for children or adolescents aged 2–18 years [15]. It measured HRQoL of the interviewees for the 1-Month period prior to the interview and consists of 4 domains: Physical Functioning (8 items), Emotional Functioning (5 items), Social Functioning (5 items), School Performance (5 items). A 5-point response scale is used in the report (0 = never a problem; 1 = almost never a problem; 2 = sometimes a problem; 3 = often a problem; 4 = almost always a problem). The scores for PedsQL all ranged from 0 to 100, and higher scores indicate better HRQoL [16]. And more specifically, physical dysfunction refers to running deficiency, and self-care inability, emotional dysfunction refers to experience of depression and anxiety, social dysfunction indicates their problem in making friends etc., and inferior school performance refers to learning disability etc.. The Chinese version of PedsQL was validated for Chinese children according to the study of Hao et al. [17], with the Cronbach α for all domains exceeding 0.70, while the Scale internal consistency reliability of our study ranges from 0.76 to 0.87.

### Procedure

The survey was performed by 10 professional researchers in July-August, 2013, all of who were trained in interviewing and administering questionnaires prior to investigation. All the children or adolescents were interviewed with the permission of their caregivers, and written informed consents were obtained from both caregivers and children or adolescents.

Taking village as a unit, we carried out household survey, and when we found a left-behind child or adolescent, we asked him/her to recommend other left-behind child(ren) or adolescent(s) in the same village, and then we investigated as many non-left-behind children recommended by the left-behind children or adolescents of similar age in the same village.

All the participants were asked to complete the questionnaire independently and factually. Questionnaires were completed by their caregivers if the children were younger than four years old. Interviewers were available to assist the subjects in completing the questionnaires, especially those who were under 12 and had semantic or conceptual understanding disability. After finishing the work above, the interviewers collected and checked the questionnaires to ensure that there were no missing data or logical mistakes.

### Data analysis

The data were entered using EpiData version 3.1. Since the HRQoL scores were not normally distributed, non-parametric rank sum tests were used in four subscales and total scores to investigate whether the left-behind children were bothered by poorer HRQoL. Univariate analyses were used to assess the association of HRQoL scores with their age, gender, caregiver, parents’ education level, duration of parents’ absence and the intervals of parent(s) coming back home to visit the children left behind. Multiple linear regression models were used to identify the effects of factors on HRQoL scores by calculating regression coefficients. The two-tailed P values of < 0.05 was considered to be significant, and the statistical analysis was completed using SAS version 9.1.

## Results

### The general information

A total of 1404 children were enrolled in this study, among which 1363(97.08%) completed the questionnaires and were included in the analysis. In these 1363 participants, 608 were left behind while 755 were not. The mean age of the left-behind children and their counterparts were 10.04 (SD = 5.97, range = 2-18) and 10.92 (SD = 5.74, range = 2-18) respectively. Demographic characteristics of all the participants are shown in Table 1, while demographic characteristics of the left-behind children are shown in Table 2.

**Table 1:** **Demographiccharacteristics of children and adolescents**

**Characteristic**	**Left-behind (%)**	**Non-left-behind (%)**	**Total (%)**
Children	608(44.61)	755(55.39)	1363(100)
Gender			
Boys	339(24.87)	370(27.15)	709(52.02)
Girls	269(19.74)	385(28.25)	654(47.98)
Age group(years)			
2-4	101(7.41)	102(7.48)	203(14.89)
5-7	70(5.14)	66(4.84)	136(9.98)
8-12	277(20.32)	339(24.87)	616(45.19)
13-18	160(11.74)	248(18.20)	408(29.93)
Caregiver's highest academic qualification			
Senior high school or higher	87(6.39)	14(1.03)	101(7.42)
Junior middle school	213(15.64)	144(10.57)	357(26.21)
Primary School	208(15.27)	320(23.49)	528(38.77)
Uneducated	100(7.34)	277(20.34)	377(27.68)
Father’s highest academic qualification			
Senior high school or higher	14(1.03)	13(0.95)	27(1.98)
Junior middle school	156(11.45)	165(12.11)	320(23.56)
Primary School	208(23.42)	320(26.14)	528(49.56)
Uneducated	120 (8.81)	220(16.15)	340(24.96)
Mother’s highest academic qualification			
Senior high school or higher	33(2.42)	36(2.64)	69(5.06)
Junior middle school	200(14.67)	215(15.77)	415(30.45)
Primary School	271(19.88)	321(23.55)	592(43.43)
Uneducated	103 (7.56)	184(13.50)	287(21.06)

**Table 2:** **Demographiccharacteristics of left-behind children and adolescents**

**Factors**	**Total(%)**
Caregiver	
Grandparents	479(78.78)
Other(includes uncle,aunt,mother,teacher)	129(21.22)
Father’s migrant working time(years)	
<1	176(28.95)
1-3	141(23.19)
3-5	96(15.79)
>5	195(32.07)
Mother’s migrant working time(years)	
Never leave	93(15.30)
<1	145(23.85)
1-3	175(28.78)
3-5	114(18.75)
>5	81(13.32)
Caregiver's highest academic qualification	
Senior high school or higher	87(6.39)
Junior middle school	213(15.64)
Primary School	208(15.27)
Uneducated	100(7.34)
Father’s visit interval(months)	
1-3	103(16.94)
4-6	67(11.02)
7-12	291(47.86)
>12	147(24.18)
Mother’s visit interval(months)	
1-3	110(18.09)
4-6	65(10.69)
7-12	232(38.19)
>12	108(17.76)

### The HRQoL status of the left-behind children and adolescents

The distribution of HRQoL scores was tested and the results indicated that the scores didn’t conform to the normal distribution. According to the Hotelling-T^2^ test, the left-behind children scored significantly lower on the HRQoL synthesis scores than the non-left-behind children (F = 6.14,P < 0.05) . And Table 3 contains the comparison of Median and Interquartile Range of PedsQL scores between the left-behind children/adolescents and the non-left-behind ones. Obviously, according to Kruskal-Wallis test HRQoL scores on Physical Functioning, Emotional Functioning and Social Functioning were significantly lower for the left-behind children than for their counterparts.

**Table 3:** **Rank sum tests for the HRQoL score of left-behind children or adolescents and the non-left-behind**

	**Left-behind (M, Q)**	**Non-left-behind (M, Q)**	**H**	**P value**
Physical summary	75.00, 21.88	81.25, 18.75	33.18	<0.0001
Emotional functioning	70.00, 25.00	70.00, 25.00	24.99	<0.0001
Social functioning	75.00, 25.00	80.00, 25.00	12.24	0.0005
School performance	70.00, 20.00	70.00, 25.00	1.11	0.2925
Average	73.91, 18.21	77.17, 17.39	29.98	<0.0001

Note: M = Median, Q = Interquartile Range.

Mean score of theleft-behind on Physical summary:75.11, mean score of the non-left-behind on Physical summary:78.55.

Mean score of the left-behind on Emotional functioning:66.91, mean score of the non-left-behind on Emotional functioning:72.15.

Mean score of the left-behind on Social functioning:77.27, mean score of the non-left-behind on Social functioning:80.52.

Mean score of the left-behind onAverage:73.04, mean score of the non-left-behind on Average:75.32.

### Univariate analyses of the left-behind children’s/adolescents’ HRQoL

According to the Hotelling T^2^ test for the left-behind children/adolescents, their HRQoL synthesis scores were found to be significantly associated with gender, age, father’s education level, duration of parents’ absence and the intervals of mother coming back home to visit the children/adolescents (Tables 4 and 5). In comparasion with the boys, the girls were more likely to be bothered by poorer HRQoL. Children aged between 2 to 4 were at remarkably higher risk of lower HRQoL synthesis scores, while children aged 8 to 12 showed a better HRQoL scores in school performance subscale. The univariate analyses showed that the left-behind children/adolescents whose father had never been to school had poorer HRQoL scores in Social Functioning subscale. It was also noteworthy that the left-behind Children whose parents were away to work in urban place for 3 to 5 years (since children birth) showed a poorer HRQoL scores than those whose parents were away for more than 5 years in Physical Functioning, Social Functioning and School Functioning. The children/adolescents whose mother came back home once every 3 to 6 months were associated with poorer HRQoL scores in Physical Functioning, Emotional Functioning and Social Functioning.

**Table 4:** **Hotelling-T2 Test of left-behind children's/adolescents’ synthesisHRQoL score**

**Factors**	**Physical summary**	**Emotional functioning**	**Social functioning**	**School performance**	**T** ^**2**^	**P**
	**(X ± SD)**	**(X ± SD)**	**(X ± SD)**	**(X ± SD)**		
Gender					3.59	P < 0.05
Boys	75.33 ± 15.77	68.18 ± 17.11^a^	76.78 ± 17.79	64.84 ± 23.78^b^		
Girls	74.76 ± 14.72	65.34 ± 17.10^b^	77.70 ± 17.65	68.94 ± 21.43^a^		
Age group(years)					27.32	P < 0.05
2-4	64.73 ± 18.64^c^	69.49 ± 19.17	75.69 ± 18.39	38.56 ± 31.09^c^		
5-7	71.83 ± 14.00^b^	68.51 ± 14.82	73.29 ± 18.37	67.64 ± 19.48^b^		
8-12	77.88 ± 12.75^a^	66.23 ± 16.86	79.15 ± 16.62	74.15 ± 14.53^a^		
13-18	78.18 ± 14.58^a^	65.82 ± 17.21	76.44 ± 18.56	70.97 ± 15.32^ab^		
Father’s highest academic qualification					2.20	P < 0.05
Uneducated	71.21 ± 14.04	62.56 ± 15.76	60.71 ± 23.69^b^	67.50 ± 12.36		
Primary school	74.98 ± 14.37	66.63 ± 17.77	77.68 ± 18.72^a^	65.74 ± 24.21		
Juniormiddle school	74.61 ± 15.24	67.01 ± 16.80	77.15 ± 17.11^a^	64.55 ± 23.79		
Senior high school or higher	76.90 ± 16.72	67.60 ± 17.55	78.58 ± 16.45^a^	73.33 ± 17.79		
Father’s migrant workingtime(years)					3.45	P < 0.05
<1	76.79 ± 13.33^a^	65.04 ± 16.21	78.41 ± 16.10^a^	73.09 ± 16.92^a^		
1-3	76.77 ± 14.58^a^	67.87 ± 16.57	78.94 ± 17.34^a^	66.83 ± 22.07^b^		
3-5	71.06 ± 16.87^b^	69.66 ± 16.82	72.71 ± 18.05^b^	61.93 ± 24.36^b^		
>5	74.28 ± 16.32^ab^	66.59 ± 18.42	77.03 ± 18.94^a^	63.05 ± 25.90^b^		
Mother’s migrant workingtime(years)					4.58	P < 0.05
Never leave	77.33 ± 14.00^a^	68.99 ± 15.11	81.72 ± 13.90^a^	68.17 ± 22.80^b^		
<1	78.05 ± 12.89^a^	66.07 ± 17.27	78.83 ± 17.65^ab^	74.03 ± 14.25^a^		
1-3	75.66 ± 15.65^a^	65.45 ± 17.49	75.31 ± 18.20^bc^	64.91 ± 23.23^b^		
3-5	68.36 ± 16.65^b^	69.32 ± 15.63	71.11 ± 16.30^c^	57.41 ± 27.73^c^		
>5	73.34 ± 16.51^a^	66.46 ± 18.70	77.17 ± 19.39^ab^	63.31 ± 25.34^b^		
Mother’s visit interval(months)					2.45	P < 0.05
0	77.33 ± 14.00^a^	68.99 ± 15.11^a^	81.72 ± 13.90^a^	68.17 ± 22.80^ab^		
1-3	78.52 ± 13.67^a^	68.66 ± 17.31^a^	80.73 ± 17.75^a^	72.82 ± 15.39^a^		
4-6	70.29 ± 16.70^b^	61.86 ± 15.42^b^	73.08 ± 17.93^b^	68.38 ± 17.10^ab^		
7 -12	73.48 ± 16.20^ab^	66.64 ± 18.46^ab^	74.78 ± 17.90^b^	63.41 ± 25.84^b^		
>12	75.95 ± 14.14^a^	67.05 ± 16.35^ab^	77.31 ± 18.89^ab^	65.00 ± 24.24^ab^		

Note: a,b,c stands for statistical significance by SNK. SNK(Student-Newman-Keuls test) was used to detect whether there were significant differences among HRQoL score levels, and whether there was a significant difference in different subgroups. More details will be found in Paulson D S. Applied statistical designs for the researcher[M]. CRC Press, 2003.

**Table 5:** **ANOVA for the HRQoL score in four domains of left-behind childrenand adolescents**

**Factors**	**Physical summary**		**Emotional functioning**		**Social functioning**		**School performance**	
	**F**	**P**	**F**	**P**	**F**	**P**	**F**	**P**
Gender	0.27	0.7854	1.94	0.0531	0.53	0.4673	-1.52	0.1301
Age group	24.23	<0.0001	1.31	0.2689	2.54	0.0557	4.39	0.0046
Father’s highest academic qualification	0.96	0.410	0.41	0.748	4.58	0.004	2.47	0.061
Father’s migrant work time	3.49	0.016	1.68	0.170	2.73	0.043	2.13	0.096
Mather’s migrant work time	6.42	<0.0001	1.02	0.397	5.06	0.001	2.05	0.086
Mather’s visit interval	4.19	0.002	1.90	0.109	4.86	0.001	0.71	0.587

### Multiple-factor analysis of the left-behind children’s HRQoL

The multivariate linear regression model revealed that HRQoL of the left-behind children was significantly associated with their age, gender, parents’ migrant working time, parents’ visit interval, and mother’s final academic qualification (Table 6). HRQoL average scores were positively associated with children’s age (β = 0.36, P = 0.004) and negatively associated with mother’s migrant working time (β = −1.02, P = 0.006). HRQoL for physical functioning increased along with age (β = 1.10, P < 0.001) and father’s visit interval (β = 1.47, P = 0.017), while it decreased along with mother’s migrant working time (β = −1.56, P < 0.001). Mother’s final academic qualification (β = 1.94, P = 0.033) was positively associated with children’s HRQoL in emotional functioning. As to social functioning, HRQoL was higher among children raised by grandparents (β = −5.49, P = 0.014), compared to that for the children raised by others (including uncle, aunt, mother, or teacher), and it was interesting that prolonged mother’s visit interval (β = −3.45, P < 0.001) and shortened father’s visit interval (β = 2.42, P = 0.004) had negative effect on children’s HRQoL. In school performance, girls had higher HRQoL scores compared to boys; besides, younger age and father’s migrant working time (β = −2.20, P = 0.002) were negatively associated with HRQoL of children.

**Table 6:** **Multivariate linear regression for factors on HRQoL scores**

**Variables**	**Physical Functioning**		**Emotional Functioning**		**Social Functioning**		**School Performance**		**Averagescores**	
	**β(SE)**	**P**	**β(SE)**	**P**	**β(SE)**	**P**	**β(SE)**	**P**	**β(SE)**	**P**
Intercept	64.02(1.53)	<0.001	71.16(2.02)	<0.001	84.86(3.78)	<0.001	45.32(3.91)	<0.001	71.46(1.58)	<0.001
Age (years)	1.10(0.14)	<0.001	-	-	-	-	2.20(0.20)	<0.001	0.36(0.12)	0.004
Mother’s migrant working time(years)	-1.56(0.44)	<0.001	-	-	-	-	-	-	-1.02(0.37)	0.006
Father’s visit interval(months)	1.47(0.62)	0.017	-	-	2.42(0.84)	0.004	-	-	-	-
Mother’s highest academic qualification	-	-	1.94(0.91)	0.033	-	-	-	-	-	-
Caregiver	-	-	-	-	-5.49(2.22)	0.014	-	-	-	-
Mother’s visit interval(months)	-	-	-	-	-3.45(0.76)	<0.001	-	-	-	-
Gender	-	-	-	-	-	-	3.45(1.69)	0.042	-	-
Father’s migrant working time(years)	-	-	-	-	-	-	-2.20(0.70)	0.002	-	-

## Discussion

This study is the first one to examine HRQoL of the left-behind children in Midwest China with PedsQL. Our findings show that the left-behind children scored significantly lower on Physical Functioning, Emotional Functioning and Social Functioning of PedsQL than the non-left-behind ones. The findings in this study correspond to the results reported in many related studies [18-20]. In contrast to the result reported by Jia ZB et al. [8], mean school performance subscale scores of the left-behind children/adolescents and the non-left-behind in this study did not differ significantly. This is probably due to socioeconomic differences between surveyed areas--Jia ZB’s survey was conducted in Shandong, which is an economically developed coastal province in Eastern China. In Shandong, most parents, especially those with good socioeconomic backgrounds, place great importance on education due to fierce competition in entering a good high school or university thus the children/adolescents from migrating families, most of which are less well-off and probably do not value education that much or cannot supervise their children due to migration, showed poorer school performance in Jia ZB’s report. However the areas we investigated are inland provinces, most of which are economically developing or undeveloped provinces, and the locals may not attach as much importance to education as those in developed areas, explaining the result that the influence of parents’ migration on school performance of the left-behind children/adolescents and the non-left-behind is less significant.

Our finding corroborates previous reports [8,21,22] that as age increased, the average HRQoL scores of the left-behind children or adolescents were significantly improved. Many studies confirm that children’s interpersonal interaction center gradually shifts to their peers from their parents as their age increases. Moreover, they become more independent and extrovert than before. All these changes improve their HRQoL. At the same time, although younger children are more vulnerable to behavior-externalizing problems [23,24], the relationship between age and mental health is still unclear. Moreover, adolescence is a special phase in which children are more vulnerable to emotional problems [25]. Thus, attention also needs to be paid especially to the mental health of adolescents.

The multivariate linear regression model for Physical Functioning shows that as age increases, HRQoL scores of the left-behind children significantly improves. The reason for this is the increasing evidence that younger children have higher risk for injuries than older children [26]; Moreover, the left-behind group are more prone to accident, which indicates that the left-behind children, especially the younger ones, need more protection and care. In addition, some researchers [20,27] reported that the left-behind children’s daily intake was notably lower than that of their counterparts. Thus, it is still a huge challenge for government to improve the living condition of the left-behind children or adolescents.

The length of mother’s absence from children or adolescents (since children birth) and visit interval were associated with average score, Physical Functioning, and Social Functioning HRQoL. It is widely acknowledged that mother plays a crucial role in fostering children, and mother’s absence due to migration for work may have negative effects on children’s daily life, for example, a child may not access breast feeding if his/her mother leaves home several months after the baby is born. Many related studies reported that mother’s care affected children’s mortality rate directly [28], and children with migrant parents were more prone to health problems compared to those in only father’s absence. But a study [29] reported that it was the length of time of father’s absence from children (since children birth) rather than that of mother’s that had more influence on children’s health; This may have to do with the patriarchal gender ideologies in rural China——children/adolescents living with father may have a better sense of safety, which in turn may promote their development. All of the above considered, if parents’ migration is unavoidable, bringing up children or adolescents with them around might be a better choice.

It is an interesting finding that children or adolescents whose father came back home more frequently displayed poorer HRQoL scores, indicating mental resilience could release negative mood [30]. The left-behind children or adolescents may adapt to the long-time separation from parents. In our study, as the time of father’s visit interval got prolonged, HRQoL scores increased correspondingly, which indicates mental resilience could be at work, but further study is needed to confirm this.

Although the previous studies [31] showed that children brought up by grandparents or only by mother were more likely to have learning hindrance, our study found that children brought up by grandparents were more likely to have a higher HRQoL than those brought up by other people such as the uncles. The reason for this might be that the supervision from other people is not careful enough and cannot make up the supervision gap out of school. In addition, other generation seldom takes care of both psychological needs and demands of personality development of children or adolescents in most instances, which may either spoil the children or fail to give them enough care [32]. While the left-behind children face many challenges in school or daily life due to their particularity in the personality shaping phase, children are vulnerable to anxiety [33] if these contradictions are neglected, which will hinder the development of mental health and lead to learning ability. This finding also needs further proof.

Our finding also shows that compared to boys, girls were more likely to suffer lower overall HRQoL but showed higher score in School Functioning. It corresponds to the finding in previous research that boys showed more interpersonal problems (such as misbehavior, reduced social interest, and disobedience) in study compared with girls, while girls showed more negative self-esteem [34]. In China, girls often need to bear more housework in a family in rural areas, especially the left-behind girls. They may be more susceptible to anxiety and depression because of the feeling of unfair treatment and being less loved due to patriarchal gender ideologies. A study reported that father’s education level was positively associated with girl’s well-being in rural China [35], indicating that better educated fathers may value their daughters’ development more, which just reversely proves the negative effects of patriarchal gender ideologies on girls in rural areas.

‘Care for and protection of the child separated from his/her parents should only be in the best interest of the child’ is enshrined in the United Nations Convention on Rights of the Child (CRC) (1989), which emphasizes that children are entitled to wide sets of rights in the same manner as adults. However, many children left behind all over the world seem to get their right of possessing healthy physical and psycho-social status overlooked. In many other developing countries like China, children have to suffer separation from their parent(s) due to labor exporting, for example left-behind children in the Caribbean are faced with risks of abuse, on top of the pains from separation from parent(s) when it comes to their psycho-social well-beings (feelings of abandonment, low self-esteem, anger, depression, material obsession and violence) and educational functioning (increased responsibilities at home, lack of affordability, motivation and parental support) [4]. It is interesting that the left-behind children were less likely to have emotional disorders compared to their counterparts in Philippines, and the reasons for that might be that the civil society has taken actions to protect the left-behind group due to its long-established labor migration. In south Asia, children of Indonesia, Vietnam and Thailand who have migrant father show emotional disorders or conduct disorders [36], which may be the result of patriarchal gender ideologies in these countries just like China. There are a substantial number of children who have been left behind as a result of parental migration in Mexico, thus the Mexican Institute of Family and Population Research developed a model to help the left-behind children and their caregivers. Not only life skill training but also method to overcome psychosocial barriers (such as shame, fears, guilt) are included in the knowledge-acquisition program, which may improve the physical and psycho-social well-beings of the children left behind and serve as an example for other countries [37].

Just as in many countries mentioned above, in China, most areas are undergoing extensive industrialization and urbanization, which attracts a number of rural laborers into developing or developed cities, causing the hot button “left-behind” issue. It is imperative that corresponding laws or regulations be introduced and enforced to harness and curb this urgent problem. On the one hand, since “being left-behind” is an undeniable negative factor for the hindered development of children, as demonstrated in this study and many previous studies, the first priority is trying our best to avoid the “left-behind” phenomenon in the first place. Most migrant workers do not take their children with them because their children cannot get access to the primary education in the city where they work due to the “HuKou” policy. In Mainland China, “HuKou”, something like internal passport, is an unavoidable formidable obstacle for those migrant workers and their children, without which people cannot enjoy the local public resources, including elementary education. If the government could guarantee all the children’s access to schooling according to where the children live rather than where their “HuKou” belongs, more migrant workers may take their children with them and the negative effect brought about by “being left-behind” may be alleviated to some extent. Some pioneer cities in China like Shanghai have conducted a pilot program which allows the migrants’ children to attend school even if they do not have local “HuKou”. Experience from the pilot program in these cities should be summed up and applied to other areas. Moreover, subsidy for childcare should be provided to those migrant workers. Raising one or more children in the metropolis is unaffordable to the majority of migrant workers who come from rural areas; this may be the other reason why children cannot be taken with their parent(s) [38]. Thus improving rural migrant worker’s wages or issuing subsidies to them could contribute to the better well-being of the left-behind children. On the other hand, if the “left-behind” situation is still unavoidable for some families, special targeted policies should be made to relieve the adverse effects deriving from children’s living separated from their parent(s). For example, government could set up special paid home leave for rural migrant workers, or build special schools for the left-behind children, which could take care of both physical and psychological well-being of these children. The government could also learn the successful experience from Mexico, and develop a model to help the left-behind children and their caregivers in line with the Chinese culture [37].

The results of this study should be viewed in light of some limitations. We just adopt convenience sampling or snowball sampling in the villages of these six provinces chosen. But this might be made up for by large sample size to some extent. Furthermore, if we combine qualitative study with quantitative research, the result could be more comprehensive. And the research does not include the survey about the parents of these left-behind children, who might have different perspectives about the situation of the left-behind children. This part is projected to be involved in further researches.

## Conclusions

In summary, our study provides preliminary evidence that the HRQoL of the left-behind children is significantly lower than that of the non-left-behind children in Midwest China. Therefore, taking preventive actions to develop right behaviors and improve health status of these children is necessary. Our findings have implications for preventive policies and programs to improve the HRQoL of the left-behind children or adolescents in China and other developing countries facing similar “left-behind” issues. All in all, whether the left-behind children grow up healthily or not directly affects the future of society, and it is a core issue that needs to be addressed in more researches.

## Additional files

Additional file 1:
**Ethics approval.**
Additional file 2:
**Informed consent.**

## Abbreviations

HRQoL: Health-related quality of life
PedsQL: Pediatric quality of life inventory
UNICEF: United Nations Children’s Fund
CRC: United Nations Convention on Rights of the Child
