# Supplementary material for: Health-related quality of life of the rural-China left-behind children or adolescents and influential factors: a cross-sectional study
Source: Health Qual Life Outcomes. 2015 Feb 27;13:29. doi: 10.1186/s12955-015-0220-x (PMC4349722; doi:10.1186/s12955-015-0220-x)
Supplement: Additional file 1: — Ethics approval. [file 12955_2015_220_MOESM1_ESM.pdf]

# The Ethics Committee of

# ChongQing Medical University

# Approval Notice

## Reference Number: 2013037

**Principal Investigators:** Xiaoni Zhong

**Title of Projects:** Health-related quality of life of left-behind children and influential factors in rural China

**Institute:** Chongqing Medical University

**Date Submitted:** 2013.06.05

**Date Reviewed:** 2013.06.12

**Date Approved:** 2013.06.12

---

**The Ethics Committee of Chongqing Medical University has reviewed the proposed use of animals in the above mentioned project. The species, strains, grade, specification and number of the animals to be used are justified. Appropriate animal care throughout the experiment, including an anesthetics, sedatives should be used. Disposition of animals at end of study, euthanasia criteria and method is accordance with the code of practice for the care and use of animals for scientific purposes. We approve the project implementation according to plan.**

重庆医科大学
医学研究伦理委员会

**The Ethics Committee of Chongqing Medical University**

**2013-06-12**
