# Supplementary material for: Health-related quality of life of the rural-China left-behind children or adolescents and influential factors: a cross-sectional study
Source: Health Qual Life Outcomes. 2015 Feb 27;13:29. doi: 10.1186/s12955-015-0220-x (PMC4349722; doi:10.1186/s12955-015-0220-x)
Supplement: Additional file 2: — Informed consent. [file 12955_2015_220_MOESM2_ESM.pdf]

Informed consent

Information sheet

Title of project：
Health-related quality of life of left-behind children or adolescents and influential factors in rural China: a cross-sectional study

General information
Health-related quality of life (HRQoL) is a good indicator in evaluating individual physical and psychological situation, which includes the status of physical, psychological and social interactions of a person's objective assessments. While China's national explosive economic growth in recent years lead to a boom in the number of left-behind children or adolescents whose HRQoL are not well studied. We are going to conduct an investigation to learn the status and influential factors of HRQoL among children in rural areas of Chongqing Municipality, Sichuan Province, Xinjiang Uygur Autonomous Region, Hunan Province, Jiangxi Province and Inner Mongolia Autonomous Region. Information gained from this study will help the public know more about left-behind children in China.

Procedures and possible risks
If you agree to participate in this project, the investigator will give you a questionnaire and explain it in details. You can fill in the questionnaire by yourself or ask the investigator for help if you had any problems or questions regarding the questionnaire.
All the processes is safe.

Possible benefits
The information will help the public know more about left-behind children in China.

Do you have to take part?
You do not have to take part in this project unless you want.

Will your personal details be confidential?
Yes. All your personal details will remain confidential and will not appear in any papers that result from this project. You have the right to access personal data and known study results.

Contacts
Should you have any concerns about the project, please do not hesitate to contact Professor Zhong Xiao-ni, School of Public Health, and Viral Hepatitis Research Institute, Chongqing Medical University, on (+86)133-0836-8059.

Consent form

Project title:
Analysis on health-related quality of life of left-behind children or adolescents and influential factors in rural China

I ______________________ (Name of subject) hereby consent to participate in the study entitled **Health-related quality of life of left-behind children or adolescents and influential factors in rural China: a cross-sectional study.**

I have read and understood the information about this study that was given/read to me. I understand that the purpose of this study is to investigate the living status and analyze the influential factors of health-related quality of life (HRQOL) among left-behind children or adolescents in rural China. I understand that in this study, what I need to do is filling in a questionnaire. It does not contains any risks in the whole process and my personal details will remain confidential and will not appear in any papers that result from this project. I have the right to access personal data and known study results.

I have been given the opportunity to ask questions about this study, and they have been answered to my satisfaction.

I consent to participate in this study and understand that I have the right to withdraw at any time.

Subject's signature:_________________ Subject's name: ________________

Investigator's signature:_______________ Investigator's name: _____________

Witness'signature: ___________________ Witness'name:__________________

Date: __________________________

知情同意书

说明书

研究课题

农村留守儿童健康相关生存质量调查及其影响因素研究

简介

健康相关生存质量(health related quality of life，HRQoL)是评估个体生理和心理状况的良好指标，它包括躯体健康、心理健康、社会交往等多方面的内容。国内留守儿童的日益增多，但其健康相关生存质量现状还不甚明确。因此本研究欲调查重庆市、四川省、新疆维吾尔族自治区、湖南省、江西省及内蒙古自治区等六个省（市）的农村留守儿童儿童及非留守儿童，以了解其健康相关生存质量现状及影响因素。其结果将有利于人们对留守儿童生活现状的了解。

过程及可能的危险

如果你同意参加该研究，调查员将会向您发放一份问卷，并向您解释问卷填写方法。您可以自行填写问卷。如对问卷理解有困难，您可以在调查员的帮助下完成问卷填写。

全过程无任何危险。

可能好处

研究结果将有助于人们对于留守儿童健康相关生存质量现状的了解。

你是否一定要参加？

除非您自愿，您可以不参加该研究。

您的个人资料是否会得到保密？

会，您的个人信息及资料将得到保密，并且不会在任何场合公开。

联系人

您如果对该研究有任何疑问，请向钟晓妮教授咨询，电话：（+86）133-0836-8059

同意书

研究课题

农村留守儿童健康相关生存质量调查及其影响因素研究

我________________(受调查者姓名)在此统一参加题为《农村留守儿童健康相关生存质量调查及其影响因素研究》的研究。

我已阅读并明白上述说明书，同意参加该研究。我知道该研究的目的**旨在探索农村留守儿童健康相关生存质量现状及其影响因素**。我知道在该研究中，**我将完成一份问卷，整个过程不包含任何风险，我的个人信息及资料将得到保密**。

我有机会向该项研究进行咨询，并得到满意答复。

我同意参加该项研究，亦同意调查者使用我的问卷信息进行研究。我知道我随时可以拒绝参加该调查。

被采访者签名________________	被采访者姓名________________
调查者签名　________________	调查者姓名　________________
见证人签名　________________	见证人姓名　________________

日期：________________
